# O-ARM navigation in tubular retractor-assisted minimal invasive parafascicular approach: technical note

**DOI:** 10.1093/jscr/rjae036

**Published:** 2024-08-07

**Authors:** Oktay Genel, Sally Price, Francesco Marchi, Ali Elhag, Oliver WroeWright, Ana Mirallave-Pescador, Steven Bibby, Keyoumars Ashkan, Francesco Vergani, Ranjeev Bhangoo, José Lavrador

**Affiliations:** School of Medicine, King’s College London, London SE1 1UL, United Kingdom; Department of Neurosurgery, King’s College Hospital Foundation Trust, London SE5 9RS, United Kingdom; Department of Neurosurgery, King’s College Hospital Foundation Trust, London SE5 9RS, United Kingdom; Department of Neurosurgery, Neurocenter of Southern Switzerland, Ente Ospedaliero Cantonale, Lugano 6900, Switzerland; Department of Neurosurgery, King’s College Hospital Foundation Trust, London SE5 9RS, United Kingdom; Department of Neurosurgery, King’s College Hospital Foundation Trust, London SE5 9RS, United Kingdom; Department of Neurosurgery, King’s College Hospital Foundation Trust, London SE5 9RS, United Kingdom; Department of Neurophysiology, King’s College Hospital Foundation Trust, London SE5 9RS, United Kingdom; Department of Neuroradiology, King’s College Hospital Foundation Trust, London SE5 9RS, United Kingdom; Department of Neurosurgery, King’s College Hospital Foundation Trust, London SE5 9RS, United Kingdom; Department of Neurosurgery, King’s College Hospital Foundation Trust, London SE5 9RS, United Kingdom; Department of Neurosurgery, King’s College Hospital Foundation Trust, London SE5 9RS, United Kingdom; Department of Neurosurgery, King’s College Hospital Foundation Trust, London SE5 9RS, United Kingdom

**Keywords:** minimally invasive, brain tumour, intra-operative, O-arm

## Abstract

Trans-sulcal minimally invasive parafascicular surgery is an emerging technique to approach deep lesions with minimal brain retraction. Localization of the tubular retractor during surgery is critical, and intraoperative magnetic resonance imaging and neuronavigation present limitations. We describe the intraoperative use of O-Arm® coupled with pre-operative tractography to precisely localize the tubular retractor. With air acting as contrast, the tubular retractor was localized in three dimensions, without any additional disruption to white matter tracts or nearby vascular structures. We conclude that visualization of tubular retractor using an intraoperative computerized tomography scan is a safe and feasible adjunct in resection of deep lesions via a minimally invasive approach.

## Introduction

Deep-seated brain lesions pose considerable challenges for surgical resection. Endoscopic approaches have been proposed to minimize brain retraction and associated vascular injury, but display limited manoeuvrability for larger lesions. Trans-sulcal minimally invasive parafascicular surgery (tsMIPS) is an emerging technique allowing safer access to deep brain lesions, including tumours, which may not be amenable to surgical treatment beyond biopsy [1, 2]. This approach minimizes disruption of the subcortical white matter tracts and injury to adjacent structures by distributing pressure circumferentially [3, 4]. Accurate intraoperative visualization of the port is essential for successful resection.

Intraoperative magnetic resonance imaging (iMRI) is a safe and accurate imaging modality for real-time tubular retractor localization [5]. Similarly, intraoperative ultrasound is a useful adjunct to maximize resection during tsMIPS [6]. However, their use may be limited by financial considerations and operator experience. Intraoperative computerized tomography (CT) imaging is more widely available and is known to be safe in brain tumour surgery [7].

Here, we describe a case of intra-operative localization of a tubular retractor with CT imaging and fusion with pre-operative tractography in a patient with an intraventricular lesion.

## Case report

**Figure 1:**
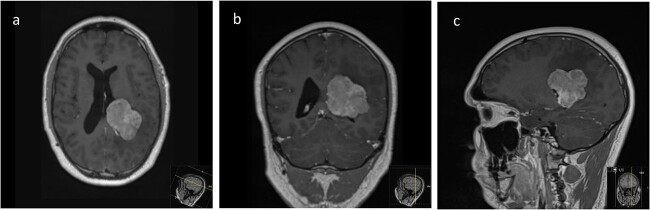
Pre-operative axial (a), coronal (b), and sagittal (c) T1-weighted post contrast MRI sequences demonstrating the avidly enhancing lesion in the posterior horn of the left lateral ventricle, with extension into the periventricular white matter.

**Figure 2:**
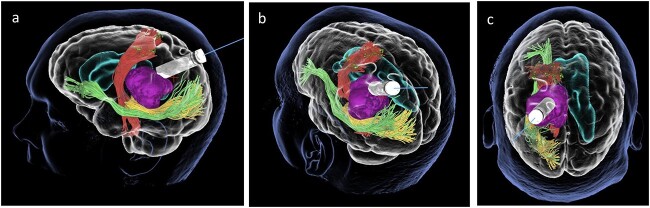
Lateral (a), ¾ lateral (b), and posterior (c) views of the pre-operative virtual 3D reconstruction model. The tumour (purple) is reached by the tubular retractor (white) and encircled by the associated white matter tracts: the CST (red) superiorly and anteriorly, and the IFOF (green) and the OR (yellow) that are located inferiorly, laterally, and posteriorly to the lesion.

We hereby present the case of a 32-year-old gentleman referred to the neuro-oncology service with an 8-months history of left sided headaches, dizziness, and blurred vision. MRI head demonstrated a 50 × 52 × 51 mm lesion in the posterior horn of the left lateral ventricle, with extension into the periventricular white matter (Fig. 1). Following multi-disciplinary discussion, a plan was made for the patient to undergo a minimally invasive approach for resection of this lesion. Pre-operative cortical and subcortical mapping was performed with navigated transcranial magnetic stimulation (nTMS) and diffusion tensor imaging (DTI). The best trajectory was defined taking into consideration the localization of the cortico-spinal tract (CST, red), the inferior fronto-occipital fasciculus (IFOF, green) and the optic radiations (OR, yellow) (Fig. 2).

**Figure 3:**
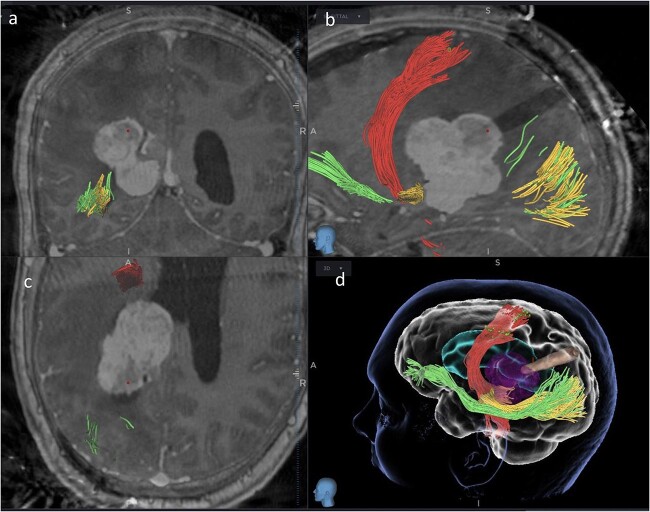
Coronal (a), sagittal (b) and axial (c) views of the merged images from intra-operative CT scans (O-Arm) with pre-operative MRI tractography. The tubular retractor is in situ. (d) Virtual 3D reconstruction of the tubular retractor (brown) in the merged images and its relationship with the tumour (purple), the CST (red), the IFOF (green), and the OR (yellow).

Subsequently, a tsMIPS approach was performed through the intraparietal sulcus. A tubular retractor (NICO BrainPath®) was cannulated 30 mm deep, directly onto the lesion. Following docking of the tubular retractor, an intra-operative CT scan (O-ARM®) was performed, and the resulting images were fused with pre-operative MRI and tractography. The port was identified at the centre of the lesion, with air acting as contrast (Fig. 3). The tumour was centrally debulked, outer sides rolled inwards, and capsule disconnected.

**Figure 4:**
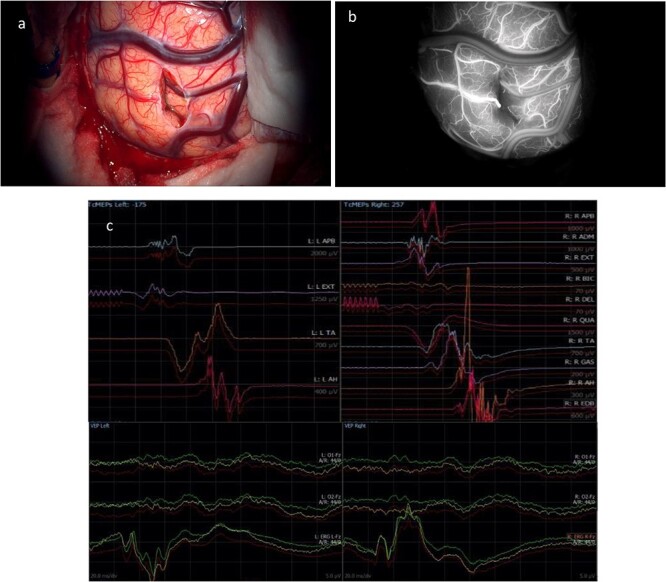
(a) Final microscopic view of the brain surface under bright light: the entry point of the operative corridor is visible at the level of the cortex, in the sulcus. (b) Microscopic final intraoperative view of the brain surface after indocyanine green injection: the vessels are represented without evidence of flow reduction or interruption. (c) Final screenshots of the intra-operative neurophysiology monitoring recorded with stable MEPs and VEPs responses (baseline in red).

Intraoperative neurophysiology monitoring (IONM) was performed throughout the case using two subdural strips, placed over the motor and occipital cortices following craniotomy. At the end of the procedure (Fig. 4a), CST was identified with subcortical stimulation at 8 mA, and both visual and motor evoked potentials (VEPs and MEPs) were stable (Fig. 4c). An external ventricular drain (EVD) was left in place. Indocyanine green angiography demonstrated no injury to nearby vascular structures (Fig. 4b).

**Figure 5:**
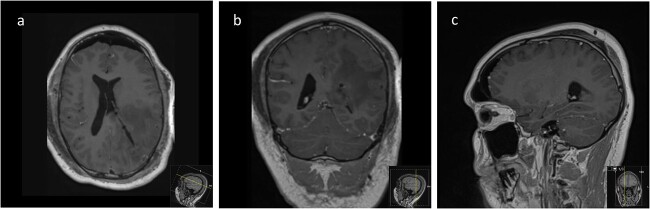
Post-operative axial (a), coronal (b), and sagittal (c) T1-weighted post-gadolinium MRI sequences of the Patient showing complete resection of the lesion with no evidence of post-operative surgical complications and complete collapse of the operative corridor pathway after removal of the tubular retractor.

The post-operative course was uncomplicated and the patient was discharged home on post-operative day 6. Histology revealed a World Health Organization (WHO) Grade I intraventricular meningioma. Immediate post-operative MRI demonstrated complete resection of this lesion (Fig. 5), with no signs of recurrence on follow-up imaging at 8 months.

## Discussion

In this case, we demonstrate the use of intraoperative CT imaging coupled with pre-operative MRI and cortical (TMS) and subcortical (tractography) brain mapping for localization of a tubular retractor during tsMIPS to an intraventricular lesion. This technique provides an accurate localization of the tubular retractor port, allowing for safe resection of this deep tumour and 3D visualization of the tubular retractor and its relationship with the lesion and the pre-operative mapping.

Pre-operative functional planning with DTI and nTMS studies are adjuncts to improve outcomes for patients with brain tumours [8]. In tsMIPS, these modalities help to define a safe sulcal entry point and appropriate trajectory for cannulation with a tubular retractor (external—cortical—and internal—subcortical—corridors). Following the insertion of the port, tumour resection is started, using regular IONM to ascertain the integrity of relevant cortical–subcortical structures, in this case, motor and visual functions. In addition, direct cortical and subcortical mapping can corroborate the pre-operative mapping information and help preserve eloquent cortical and subcortical structures. Subsequent intraoperative visualization of the approach trajectory from both anatomical and functional perspectives is paramount in guiding surgical decisions. Combining pre-operative functional studies with intra-operative anatomical imaging therefore provides a higher degree of information to safely resect eloquent lesions.

Conventional methods of neuronavigation using optical or electromagnetic neuronavigation techniques are commonly employed in neurosurgical oncology, and have been shown to improve outcomes for patients [9]. Neuronavigation is a critical adjunct in tsMIPS where it is used to control the trajectory as the retractor is pushed through the sulcus and towards the lesion [10]. Despite being useful, these modalities are limited as they only provide a unidimensional view of a location at any given time. Moreover, electromagnetic navigation systems can interfere with IONM, causing noise in the acquisition of MEPs [11]. With air acting as a contrast agent, intraoperative CT provides an additional advantage as the location of the tubular retractor can be visualized in three dimensions.

As tumours are debulked, a relatively significant degree of brain shift occurs, limiting the accuracy and extensive use of neuronavigation tools based on pre-operative imaging [12]. Intra-operative imaging with MRI, ultrasound, and CT helps to mitigate the surgical information lost due to inaccurate neuronavigation [13], but cannot be merged with pre-operative tractography with high reliability. In this regard, tubular retractors only cause a minimal degree of brain shift, as their rigid structure holds parenchyma and white matter tracts in place. Any kind of intra-operative imaging, including CT scans is thus more reliable. When compared with other intraoperative imaging modalities, intraoperative CT has some advantages and disadvantages that should be balanced according to the aims of each procedure and surgical team. When compared with MRI, CT is less expensive, requires less time to perform, has a better quality in the bone window but provides a less detailed parenchymal image. Intraoperative ultrasound is more ubiquitous when compared with CT. Despite being less expensive and easier in image acquisition, ultrasound has some disadvantages. The small size craniotomies used in MIPS approach make it more challenging to use common probes to ascertain and estimate the extent of resection, particularly after brain cannulation, due to limited space available and the artefact produced by the tube itself. This is partially overcome by the burr hole probes. However, these are affected by the tubular retractor constraints that hamper the visualization around the walls of the tube. Therefore, particularly in circumstances of deep-docking for tumour resection, the ultrasound-derived information is limited and can bias the surgical team in the wrong direction. Also, the intraoperative CT provides multiplanar visualization (axial, coronal, and sagittal) that is important when operating in narrow corridors such as the ones produced by tubular retractors. These orthogonal planes are more familiar to the surgical teams when compared with the ones produced by the intraoperative ultrasound. Finally, the CT has an operator independent fusion system with pre-operative images whilst a significant number of ultrasound devices available in the market rely on human fusion to a certain degree. Despite the anchor effect of the tubular retractor in the decrease of brain shift, the consistency of the lesions and their progressive debulking may produce more significant changes in the areas around the lesion when compared with the remaining brain. Therefore, accurate intraoperative visualization techniques are paramount during tumour resection. Nevertheless, the choice amongst the available solutions depends upon the aims of the surgical teams as well as potential financial and time-related limitations.

## Conclusion

Visualization of tubular retractor localization with an intraoperative CT scan and its integration with pre-operative cortical and subcortical mapping is a feasible and safe adjunct for resection of deep-seated lesions via a tsMIPS approach that improves intraoperative visualization and guidance during this minimally invasive technique.

## Conflict of interest statement

The authors do not have any conflict of interest to declare.

## Funding

No funding has been received to perform the work presented in this manuscript.
